# Application of individualized localization technology based on multimodal image spatial coordinate mapping in suboccipital retrosigmoid approach surgery

**DOI:** 10.3389/fneur.2026.1803347

**Published:** 2026-09-22

**Authors:** Yongzhen Hu, Haitong Xu, Jianhao Liang, Zhongqi Wang, Yongkai Qin, Liyang Zhang, Jingjing Zheng, Yingchao Jing, Xuesong Li

**Affiliations:** 1 Department of Neurosurgery, The Affiliated Huizhou Hospital, Guangzhou Medical University, Huizhou, China; 2 Department of Radiology, The Affiliated Huizhou Hospital, Guangzhou Medical University, Huizhou, China

**Keywords:** 3D slicer, multimodal image fusion, precise localization, suboccipital retrosigmoid approach, transverse-sigmoid sinus junction (TSSJ), venous sinus injury

## Abstract

**Background:**

The suboccipital retrosigmoid approach is classic for cerebellopontine angle lesions, but accurate localization of the transverse sinus (TS), sigmoid sinus (SS), and transverse-sigmoid sinus junction (TSSJ) remains challenging due to anatomical variations. Traditional landmark-based localization is prone to venous sinus injury, highlighting the need for precise preoperative planning.

**Objectives:**

To evaluate the efficacy of 3D Slicer-assisted individualized localization technology based on multimodal image spatial coordinate mapping for TS, SS, and TSSJ in suboccipital retrosigmoid surgery, and to assess its value in reducing complications and optimizing surgical outcomes.

**Methods:**

A retrospective sequential before-and-after cohort analysis included 91 patients (December 2023–October 2025) divided into the experimental group (*n* = 52, 3D Slicer-aided CT/CTV fusion + 3D reconstruction) and control group (*n* = 39, traditional 2D imaging + surface landmarks). Key indicators (keyhole positioning accuracy, venous sinus injury rate, craniotomy time, intraoperative blood loss, postoperative hospital stay) were compared using R 4.5 software.

**Results:**

Baseline data were balanced between groups (all *P* > 0.05). The experimental group achieved 100% keyhole positioning accuracy (vs. 76.9% in controls, *P* < 0.001) and zero venous sinus injury (vs. 10.3% in controls, *P* = 0.031). Craniotomy time (28.12 ± 3.82 vs. 35.64 ± 5.51 min), intraoperative blood loss (64.81 ± 10.84 vs. 97.74 ± 51.03 ml), and postoperative hospital stay (7.81 ± 1.5 vs. 10.51 ± 5.9 days) were significantly improved in the experimental group (all *P* < 0.001). Learning curve analysis showed experimental group preoperative planning time declined with experience: inflection point at Case 7, 16.0% time reduction (29.29 ± 4.96 to 24.60 ± 4.14 min, *P* = 0.048), and learning rate 0.095.

**Conclusions:**

3D Slicer-assisted individualized localization is associated with improved precision of TS-SS complex positioning, reduced venous sinus injury risk, and optimized surgical efficiency. Its low-cost and user-friendly features correlate with favorable conditions for adoption within primary-hospital settings, showing promising clinical application prospects.

## 1 Introduction

The suboccipital retrosigmoid approach serves as a well-established surgical strategy for managing cerebellopontine angle lesions, including hemifacial spasm, trigeminal neuralgia, meningiomas, and acoustic neuromas. The safety and efficiency of the procedure heavily depend on the accurate identification of the inferior margin of the transverse sinus, posterior margin of the sigmoid sinus, and transverse-sigmoid sinus junction (TSSJ) during craniotomy (1–3). Conventional localization methods primarily rely on surface bony landmarks such as the “asterion”, “mastoid process”, and “superior nuchal line”. However, the anatomical structure of this region exhibits substantial individual variability, and empirical localization alone is prone to venous sinus injury, which is associated with intraoperative massive hemorrhage, prolonged surgical duration, and even adverse impacts on patient prognosis (4–6). Therefore, exploring a more precise and intuitive preoperative localization technique is clinically significant for enhancing the outcomes of suboccipital retrosigmoid craniotomy.

As an open-source medical image processing platform, 3D Slicer integrates multimodal data fusion, three-dimensional (3D) reconstruction, and visualization capabilities (7–9). It can consolidate imaging data from CT and CTV to clearly illustrate the spatial relationships of anatomical structures, addressing the limitations of traditional two-dimensional imaging. Through a retrospective case analysis, this study aims to comprehensively evaluate the application value of this technology in the individualized preoperative precise localization of the transverse sinus, sigmoid sinus, and TSSJ during suboccipital retrosigmoid craniotomy. Additionally, it seeks to assist in planning safe skull incision pathways and further analyze its clinical effects in reducing the risk of venous sinus injury, shortening surgical time, and improving perioperative outcomes.

Most prior researches adopting 3D Slicer for retrosigmoid craniotomy only realized simple multimodal image fusion and rough 3D anatomical visualization without quantifiable surface coordinate calibration. This study innovatively constructs a standardized spatial coordinate cross-mapping system based on thin-slice CT and CTV multimodal registration, which converts empirical landmark localization into repeatable quantitative geometric projection onto the skull surface. We further quantified the learning curve of this full preoperative planning workflow and proposed a low-cost, grassroots-hospital-oriented hierarchical application scheme, providing a novel replicable precise localization paradigm for cerebellopontine angle lesion surgery.

## 2 Materials and methods

### 2.1 Study design and participants

This study was designed as a retrospective sequential (before-and-after) cohort study, approved by the Ethics Committee of our hospital (Approval No.: 2023-KY-101-01), with a waiver of informed consent. The waiver was granted because the study involved retrospective analysis without additional interventions on patients, only utilizing routinely collected clinical imaging and medical records. Patient privacy was strictly protected throughout data processing, and the research carried minimal risk, complying with the requirements of the Declaration of Helsinki.

A total of 91 eligible patients were enrolled, including 52 in the experimental group (3D Slicer- assisted group) and 39 in the control group (traditional surgery group). Consecutive medical records of patients who underwent suboccipital retrosigmoid approach surgery in the Department of Neurosurgery of our hospital from December 2023 to October 2025 were collected retrospectively.

Group allocation was strictly based on the sequential implementation of clinical technical protocols in our department rather than surgeon preference, case complexity, or the availability of 3D printing equipment. Specifically, patients who received individualized localization via 3D Slicer-based multimodal image fusion and reconstruction after the official clinical application of this technology were assigned to the experimental group (*n* = 52). Patients receiving conventional localization using two-dimensional imaging combined with surface bony landmarks before the technical implementation (December 2023–November 2024) were allocated to the control group (*n* = 39). Both groups followed identical inclusion and exclusion criteria and surgical indications, and all patients were operated on by the same surgical team throughout the study period, which was designed to minimize the potential selection bias caused by non-standard allocation.

Inclusion criteria: (1) Preoperative imaging diagnosis of cerebellopontine angle lesions (e.g., trigeminal neuralgia, hemifacial spasm, meningioma, acoustic neuroma, epidermoid cyst); (2) Age between 18 and 75 years; 3) Complete clinical and imaging data.

Exclusion criteria: (1) Previous surgical history in the same region; (2) Comorbidities of severe coagulation disorders, cardiopulmonary insufficiency, or other surgical contraindications; (3) Malignant tumors with distant metastasis; (4) Concurrent other intracranial diseases requiring simultaneous treatment; (5) Missing clinical or imaging data.

### 2.2 Image processing and localization methods

#### 2.2.1 Experimental group

All patients underwent preoperative thin-slice cranial CT (imaging parameters: matrix 512 × 512, field of view 257 mm, slice thickness 0.6 mm, voltage 120 kV, current 220 mA) and 3D-CT venography (CTV) (imaging parameters: matrix 512 × 512, field of view 257 mm, slice thickness 0.6 mm, voltage 100 kV, current 257 mA). The scanning range covered from the skull vertex to the skull base to display the skull structure and venous sinus system with high resolution. Scanning data were exported in DICOM format and imported into 3D-Slicer software (version 5.0.2).

Precise registration of CT and CTV images was achieved using the General Registration or Landmark Registration module to ensure the anatomical consistency between the skull structure and the venous sinus system. The Segment Editor module was employed to segment and three-dimensionally model the skull, transverse sinus, sigmoid sinus, and TSSJ, generating a fused 3D visualization model.

After adjusting the 3D model to the surgical position, the Display function of the Model module was used to visualize key anatomical structures, focusing on the area relevant to the suboccipital retrosigmoid approach. The Four-up display mode enabled simultaneous observation of axial, sagittal, coronal, and 3D models. The Markup module was used to mark the core point S at the medial edge of TSSJ (Figure 1A). The spherical magnification function was applied to enlarge point S until it was tangent to the outer skull plate (combining 2D and 3D images in real time), and the tangent point was labeled as the keyhole point T (Figure 1B). The transparency of the skull model was repeatedly adjusted to confirm the safe distance between the burr hole point and the venous sinus, with fine-tuning of point T's position if necessary.

**Figure 1:**
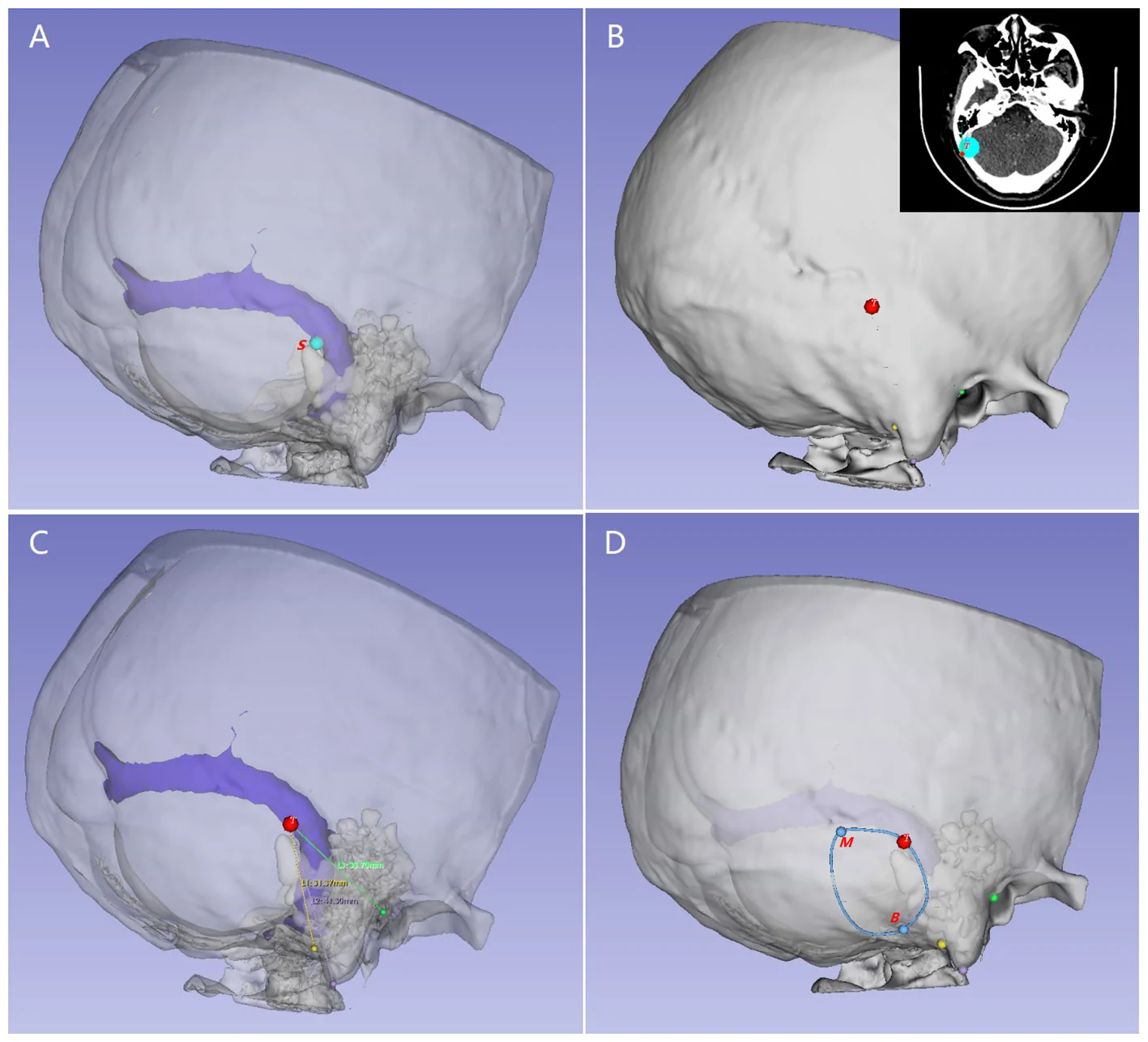
Schematic of Key Localization Steps Using 3D Slicer. **(A)** Increase skull transparency to visualize key anatomical regions, and mark the core point S (green point) at the medial edge of the transverse-sigmoid sinus junction (TSSJ) via the Markup module. **(B)** Restore the skull surface, magnify point S spherically until it is tangent to the outer skull plate, mark the tangent point as the keyhole point T (red point), and label anatomical reference points including the apex of the digastric groove (yellow point), mastoid tip (purple point), and midpoint of the posterior wall of the external auditory canal (green point). **(C)** Measure the spatial distances (L1, L2, L3) from each anatomical reference point to the keyhole point T (red point) sequentially. **(D)** Mark the bone window boundary points M and B according to the individualized selection of craniotomy window size for each patient, completing the precise planning of the bone window.

In the model, the apex of the digastric groove (yellow point), mastoid tip (purple point), and midpoint of the posterior wall of the external auditory canal (green point) were marked as anatomical reference points. The spatial distances (L1, L2, L3) from the keyhole point T to each reference point were accurately measured. A compass was used to draw circles with L1 and L2 as radii, and the intersection of the two circles was the localization of key point T on the skull surface. L3 was used for secondary verification (Figure 1C), and multiple cross-validations were performed using the spatial distances of the three reference points to further improve localization accuracy.

Meanwhile, the 3D reconstruction model intuitively displayed the course and angular relationship of the transverse sinus and sigmoid sinus, clarifying the anatomical association between mastoid air cells and the venous sinus during surgery, and providing a reference for the individualized selection of craniotomy window size. Based on the above localization logic and the patient's individual anatomical characteristics, the medial inferior edge boundary of the transverse sinus of the bone window was determined: point M was marked 3–5 cm away from the keyhole point T and the medial inferior edge of the transverse sinus. The spatial distances from any two of the 4 identified marker points to point M were measured, and the actual position of point M was confirmed via surface projection. Using the same method, point B was marked at the outer inferior edge of the bone window close to the bottom of the sigmoid sinus to complete the precise planning of the bone window boundary (Figure 1D). The simplified flowchart of individualized preoperative localization planning for each patient using 3D-Slicer is shown in Figure 2.

**Figure 2:**
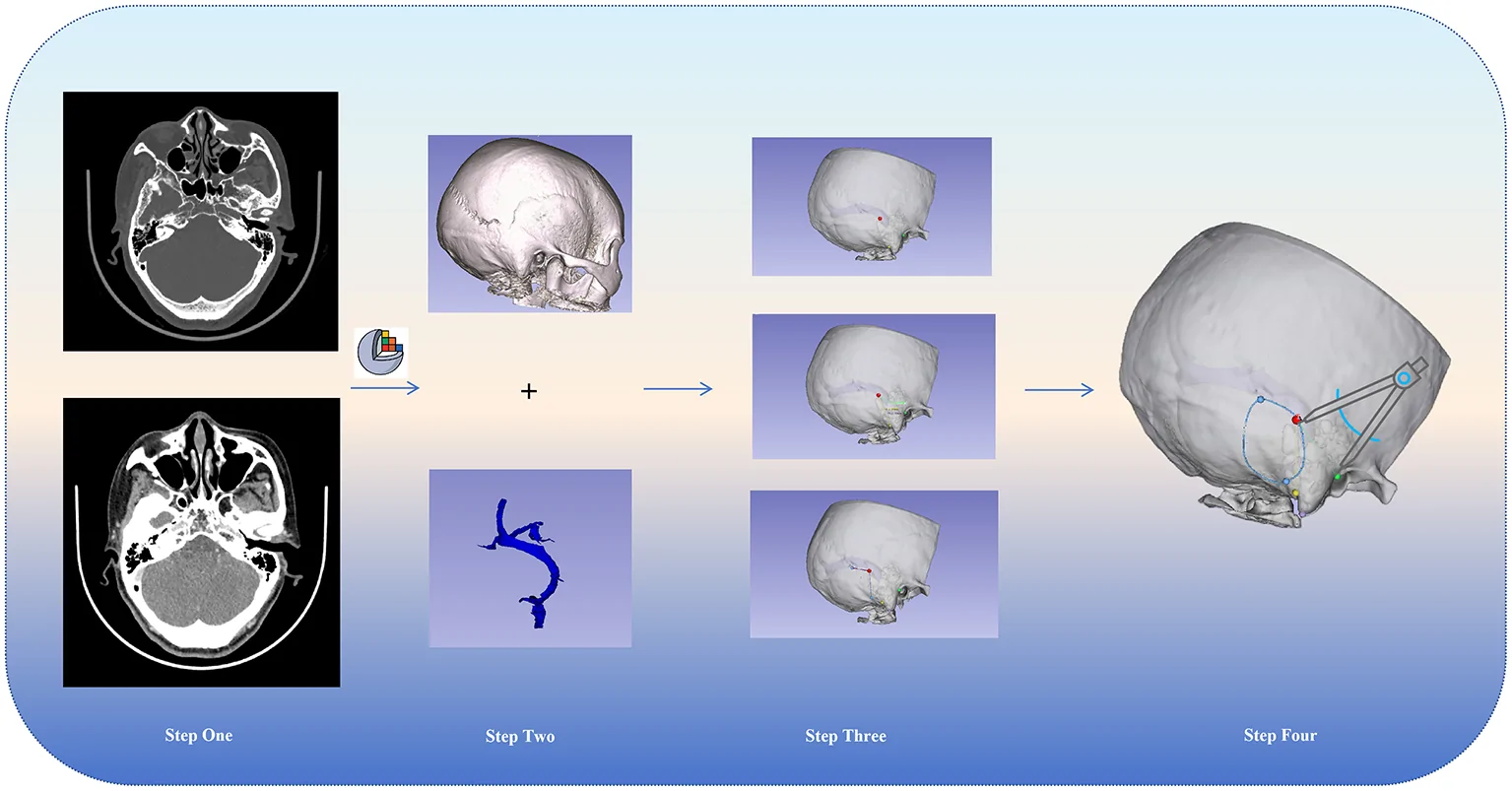
Flowchart of Preoperative Localization Planning with 3D Slicer. First step: Import and register CT and CTV images of each patient. Second step: Segment the skull and venous sinuses using the Segment Editor module in 3D Slicer. Third step: mark the core point, keyhole point, anatomical reference points, inter-point distances, and bone window range in the Markup module. Fourth step: transfer the preoperative planning parameters to the operative field, and mark as well as measure the planned points on the patient's scalp with compasses and straightedge during surgery.

To further ensure surgical precision, 1:1-scale 3D-printed transparent skull models were fabricated for some patients with complex cases. The printing material was medical photosensitive resin with a printing accuracy of ±0.1 mm. The production cycle was approximately 2–3 days (including transportation time from the factory to the hospital), and the cost per case was about 800–1,000 yuan. These models provided physical reference for intraoperative localization and allowed preoperative rehearsal.

#### 2.2.2 Control group

The conventional localization method was adopted. Combined with preoperative two-dimensional imaging data and surface bony landmarks (e.g., asterion, mastoid process), the burr hole position and the grinding range of the skull in the surgical area were determined based on the surgeon's clinical experience.

#### 2.2.3 Practical implementation requirements for this technique

The preoperative multimodal mapping workflow is jointly completed by neurosurgeons and radiologists. A standardized 2-week hands–on training program covering image import, registration, segmentation, and landmark marking is required before routine clinical adoption. Virtual preoperative planning without 3D printing typically consumes 20–30 min per patient. Required imaging hardware only includes routinely-available thin-slice cranial CT and CTV (slice thickness 0.6 mm). The 3D-Slicer software is open-source with zero licensing costs. Patient-specific 1:1 transparent 3D-printed skull models (cost 800–1,000 RMB per case, production cycle 2–3 days) are selectively indicated for complex anatomical variants, giant skull-base lesions, or cases with severely distorted venous-sinus anatomy for physical rehearsal; 3D printing is not mandatory for routine cases. For primary hospitals without in-house 3D-printing hardware, third-party commercial printing services can be utilized.

### 2.3 Surgical procedures

All surgeries were performed by the same team of physicians with extensive skull base surgery experience (all chief surgeons were associate chief physicians or above with more than 10 years of skull base surgery experience). Patients were placed in a semi-supine or lateral prone position to position the mastoid area at the highest point of the surgical field. An “S”-shaped incision was made along the posterior hairline behind the ear, and the skin, subcutaneous tissue, and neck muscles were separated layer by layer. The occipital artery was ligated or transected if necessary, and bone wax was used to block bleeding from the mastoid emissary vein. The posterior wall of the ear was separated laterally to fully expose relevant anatomical landmarks.

According to the preoperative localization results of the two groups, the keyhole points were identified. A drill was used to create a burr hole along the medial edge of the marked point until the dura mater was reached. A curved elevator was employed to separate adhesions on the dura mater surface, followed by further gentle separation with a small piece of gelatin sponge to carefully confirm the position of TSSJ. Subsequently, a high-speed craniotome was used for craniotomy, sawing the skull first toward the midline and then along the sigmoid sinus.

Requirements for the bone window boundary: the upper edge reached the inferior edge of the transverse sinus, the outer upper edge extended to TSSJ, the lateral edge was close to the sigmoid sinus, the lower edge was the horizontal part of the posterior cranial fossa, and the medial edge was 3–5 cm away from the sigmoid sinus. After craniotomy, adhesions between the dura mater and the inner skull plate were carefully separated to expose the surgical area. For some patients, a high-speed drill and Kerrison rongeur were used to resect part of the mastoid process at the medial edge of the sigmoid sinus.

### 2.4 Outcome measures

#### 2.4.1 Primary safety indicators

Accuracy of keyhole positioning: verified by postoperative thin-slice CT (same imaging parameters as preoperative) 3D reconstruction performed on the first postoperative day. *Post-hoc* distance measurements were immediately conducted by the same designated raters (one neurosurgeon and one radiologist) who performed preoperative spatial coordinate measurements to reduce inter-rater measurement error. Successful localization cases were annotated accordingly. Notably, reconstructed postoperative image projects were not permanently stored for this retrospective series. The ideal projection point was defined as the 3D projection point of the medial edge of TSSJ after postoperative reconstruction. The burr hole point was considered accurate if the 3D spatial distance from the burr hole point to the ideal projection point was < 3 mm. This < 3 mm accuracy threshold was empirically adopted based on clinical experience from functional-neurosurgical robot-assisted positioning literature, as no well-established reference threshold exists for retrosigmoid keyhole localization. The measuring tool was the Distance module of 3D-Slicer. Blinded assessment was not performed in this study.

Incidence of intraoperative venous sinus injury: defined as intraoperative venous sinus wall damage with bleeding requiring special treatment (e.g., suture, compression with hemostatic materials).

#### 2.4.2 Efficiency indicators

Craniotomy time (time from skin incision to complete exposure of the dura mater, including mastoid grinding time), intraoperative blood loss, postoperative drainage volume, and postoperative hospital stay.

### 2.5 Statistical analysis

Statistical analysis was performed using R 4.5 software. Normality of continuous data was tested by the Shapiro-Wilk test, and homogeneity of variance was assessed by the Levene test. Continuous data conforming to normal distribution and homogeneity of variance were expressed as mean ± standard deviation ($\bar{\text{x}}$ ± s), and intergroup comparisons were conducted using the independent samples *t*-test. Categorical data were presented as counts (percentages), and intergroup comparisons were performed using the χ^2^ test or Fisher's exact probability test.

Multiple linear regression models were constructed using the stats package (version 4.5.0) in R 4.5 software. Confounders including age, sex, lesion type, and anatomical variation were adjusted for in the models to evaluate the independent effect of the 3D Slicer-assisted localization strategy on continuous perioperative outcomes. *Post-hoc* power analysis for all outcome indicators was performed using the pwr package (version 1.3-0) in R 4.5 software, with a two-tailed significance level of α = 0.05. Crude and adjusted effect-size estimates together with 95% confidence intervals for primary and secondary peri-operative outcomes are summarized. The effect size was calculated based on the intergroup mean difference and the pooled standard deviation.

For the learning curve analysis of preoperative planning time, the LOESS smoothing curve (span = 0.6) was used to illustrate trend changes. Segmented regression analysis was applied to detect the inflection point of the learning curve and divide the learning stages. The exponential decay model (Time = *a* × Case^∧^(−*b*)) was used to fit the learning curve and calculate the learning rate (*b*-value). The independent samples *t*-test was used to compare time differences between different learning stages. Graphs were plotted using packages such as ggplot2, ggpubr, and segmented. All tests were two-tailed, and *P* < 0.05 was considered statistically significant.

## 3 Results

### 3.1 Baseline characteristics

A total of 91 patients were enrolled in this study, including 52 in the experimental group and 39 in the control group. No statistically significant differences were observed in baseline data between the two groups, such as age, gender, anatomical variations, and distribution of lesion types (all *P* > 0.05), indicating that the baseline characteristics were balanced and comparable (Table 1). Specifically, the mean age was 50.17 ± 7.58 years in the 3D Slicer group and 52.03 ± 7.01 years in the control group (*P* = 0.232). The predominant lesion types in both groups were trigeminal neuralgia and hemifacial spasm, with similar distribution patterns (*P* = 0.966). In the 3D Slicer experimental group, there were 28 males and 24 females, including 16 cases with anatomical variations. In the control group, there were 18 males and 21 females, with 14 cases of anatomical variations.

**Table 1:** Comparison of baseline characteristics between groups.

Variables	Total	3D Slicer	Control	*P*-Value
Age (year)	51.0 ± 7.36	50.17 ± 7.58	52.03 ± 7.01	0.232
Sex				0.607
Male	46 (50.5%)	28 (53.8%)	18 (46.2%)	
Female	45 (49.5%)	24 (46.2%)	21 (53.8%)	
Anatomical variation				0.772
Yes	30 (33.0%)	16 (30.8%)	14 (35.9%)	
No	61 (67.0%)	36 (69.2%)	25 (64.1%)	
Lesion type				0.966
Trigeminal neuralgia	32 (35.2%)	18 (34.6%)	14 (35.9%)	
Hemifacial Spasm	28 (30.8%)	15 (28.8%)	13 (33.3%)	
Meningioma	16 (17.6%)	10 (19.2%)	6 (15.4%)	
Acoustic neuroma	9 (9.9%)	5 (9.6%)	4 (10.3%)	
Epidermoid cyst	6 (6.6%)	4 (7.7%)	2 (5.1%)	

### 3.2 Localization effect and surgical indicators

The 3D Slicer group outperformed the control group in all key indicators (Table 2). Regarding surgical efficiency, the mean craniotomy time in the 3D Slicer group was significantly shorter than that in the control group (28.12 ± 3.82 min vs. 35.64 ± 5.51 min, *P* < 0.001). Intraoperative blood loss (64.81 ± 10.84 ml vs. 97.74 ± 51.03 ml, *P* < 0.001) and postoperative drainage volume (90.13 ± 11.68 ml vs. 114.18 ± 18.97 ml, *P* < 0.001) were significantly lower in the 3D Slicer group. Additionally, the postoperative hospital stay was significantly shorter in the 3D Slicer group compared with the control group (7.81 ± 1.5 days vs. 10.51 ± 5.9 days, *P* < 0.001).

**Table 2:** Comparison of perioperative outcomes between groups.

Variables	Total	3D slicer	Control	*P*-Value
Craniotomy time (min)	31.34 ± 5.93	28.12 ± 3.82	35.64 ± 5.51	< 0.001^***^
Intraoperative blood loss (ml)	78.92 ± 37.88	64.81 ± 10.84	97.74 ± 51.03	< 0.001^***^
Postoperative drainage volume (ml)	100.44 ± 19.3	90.13 ± 11.68	114.18 ± 18.97	< 0.001^***^
Postoperative hospital stay (days)	8.97 ± 4.22	7.81 ± 1.5	10.51 ± 5.9	< 0.001^***^
Accurate positioning of key holes				< 0.001^***^
Yes	82 (90.1%)	52 (100%)	30 (76.9%)	
No	9 (9.9%)	0 (0%)	9 (23.1%)	
Venous sinus injury				0.031^*^
Yes	4 (4.4%)	0 (0%)	4 (10.3%)	
No	87 (95.6%)	52 (100%)	35 (89.7%)	

^*^P < 0.05; ^***^P < 0.001.

In terms of accuracy and safety, the 3D Slicer group achieved a 100% accuracy rate in keyhole positioning, which was significantly higher than the 76.9% in the control group (*P* < 0.001). Notably, no venous sinus injury occurred in the 3D Slicer group, whereas the incidence of venous sinus injury in the control group was 10.3% (*P* = 0.031). All 4 cases of venous sinus injury in the control group were associated with inaccurate positioning, and 3 of these 4 cases (75.0%) had anatomical variations such as anteriorly positioned sigmoid sinus or inferiorly located transverse sinus.

### 3.3 Multivariate adjusted analysis

After adjusting for confounders including age, sex, lesion type, and anatomical variation, the between-group differences in perioperative outcomes remained statistically significant (Supplementary Table S1): the 3D Slicer group was associated with an average reduction of 7.20 min in craniotomy time (*P* < 0.001), an average reduction of 31.49 ml in intraoperative blood loss (*P* < 0.001), an average reduction of 23.07 ml in postoperative drainage volume (*P* < 0.001), and an average reduction of 2.60 days in postoperative hospital stay (*P* = 0.002). These results indicate that the observed association between the localization strategy and improved perioperative outcomes is independent of baseline confounders, supporting the favorable robustness of the study conclusions. Full crude and adjusted effect-size estimates together with corresponding 95% confidence intervals for all peri-operative endpoints are presented in Table 3.

**Table 3:** Comparison of primary outcomes between the two groups.

Outcome	3D Slicer (*n* = 52)	Control (*n* = 39)	Crude effect (95% CI)^†^	Crude *P*	Adjusted effect (95% CI)^*^	Adjusted *P*	*Post-hoc* power (1–β)
**Continuous outcomes**							
Craniotomy time, min	28.12 ± 3.82	35.64 ± 5.51	−7.53 (−9.58, −5.47)	< 0.001	−7.20 (−8.6, −5.79)	< 0.001	100.00%
Intraoperative blood loss, ml	64.81 ± 10.84	97.74 ± 51.03	−32.94 (−49.72, −16.15)	< 0.001	−31.49 (−43.87, −19.1)	< 0.001	99.40%
Postoperative drainage, ml	90.13 ± 11.68	114.18 ± 18.97	−24.04 (−30.93, −17.16)	< 0.001	−23.07 (−26.82, −19.32)	< 0.001	100.00%
Postoperative hospital stay, day	7.81 ± 1.50	10.51 ± 5.90	−2.71 (−4.66, −0.75)	0.008	−2.60 (−4.18, −1.03)	0.002	88.20%^?^
**Binary outcomes**							
Accurate positioning rate, *n* (%)	100% (52/52)	76.9% (30/39)	Inf (3.10, Inf)^‡^	< 0.001	Not performed^§^	NA	99.70%
Sinus injury rate, *n* (%)	0% (0/52)	10.3% (4/39)	0.00 (0, 1.09)	0.031	Not performed^§^	NA	86.80%^?^

^*^Adjusted for age, sex, lesion type, and anatomical variation via multiple linear regression.

^†^Continuous outcomes reported as mean difference (MD); binary outcomes as odds ratios (OR) from two-sided Fisher's exact test.

^‡^The OR tends toward infinity due to zero events of inaccurate positioning in the test group.

^§^Not performed: the number of events was insufficient to meet the sample size assumptions of multivariable logistic regression.

^?^*Post-hoc* power analysis demonstrated statistical power ≥ 88.2% for all continuous outcomes and ≥ 86.8% for all binary outcomes (α = 0.05, two-sided).

### 3.4 Retrospective *Post-hoc* power analysis

*Post-hoc* power analysis was performed to evaluate the sample size adequacy for all primary endpoints. All endpoints met the widely accepted 80% statistical power threshold. Specifically, the statistical power for craniotomy time, intraoperative blood loss, postoperative drainage volume, and accuracy of keyhole positioning all exceeded 99%, while the power for postoperative hospital stay and incidence of venous sinus injury was 88.2 and 86.8%, respectively. These findings confirm that the current sample size has sufficient statistical power to detect true intergroup differences, supporting the statistical robustness of the study conclusions. Detailed *post-hoc* power results are annotated in the footnotes of Table 3.

### 3.5 Learning curve analysis

Before the start of this study (i.e., Case 1), the neurosurgeon and radiologist involved in preoperative planning had no prior clinical or technical experience with the 3D Slicer software, and they only received a standardized theoretical and hands-on training course for 3D Slicer (focusing on image import, registration, segmentation, and landmark marking) with a duration of 2 weeks organized by the hospital's medical imaging center before the formal application of the technology.

With the accumulation of clinical cases, the preoperative planning time showed a gradual downward trend. Surgical planning was jointly performed by one neurosurgeon with 10 years of work experience and one radiologist with 12 years of work experience. Segmented regression combined with the CUSUM method identified an estimated inflection point of the learning curve at the 7th case (Figure 3), after which performance transitioned toward a more proficient phase. This case-7 cut-off represents a statistical estimate rather than an absolute definitive proficiency threshold. The average planning time in the early learning phase (Cases 1–7) was 29.29 ± 4.96 min, which was significantly reduced to 24.60 ± 4.14 min in the proficiency phase (Cases 8–52) (*t* = 2.38, *P* = 0.048), representing a 16.0% reduction (Figure 4).

**Figure 3:**
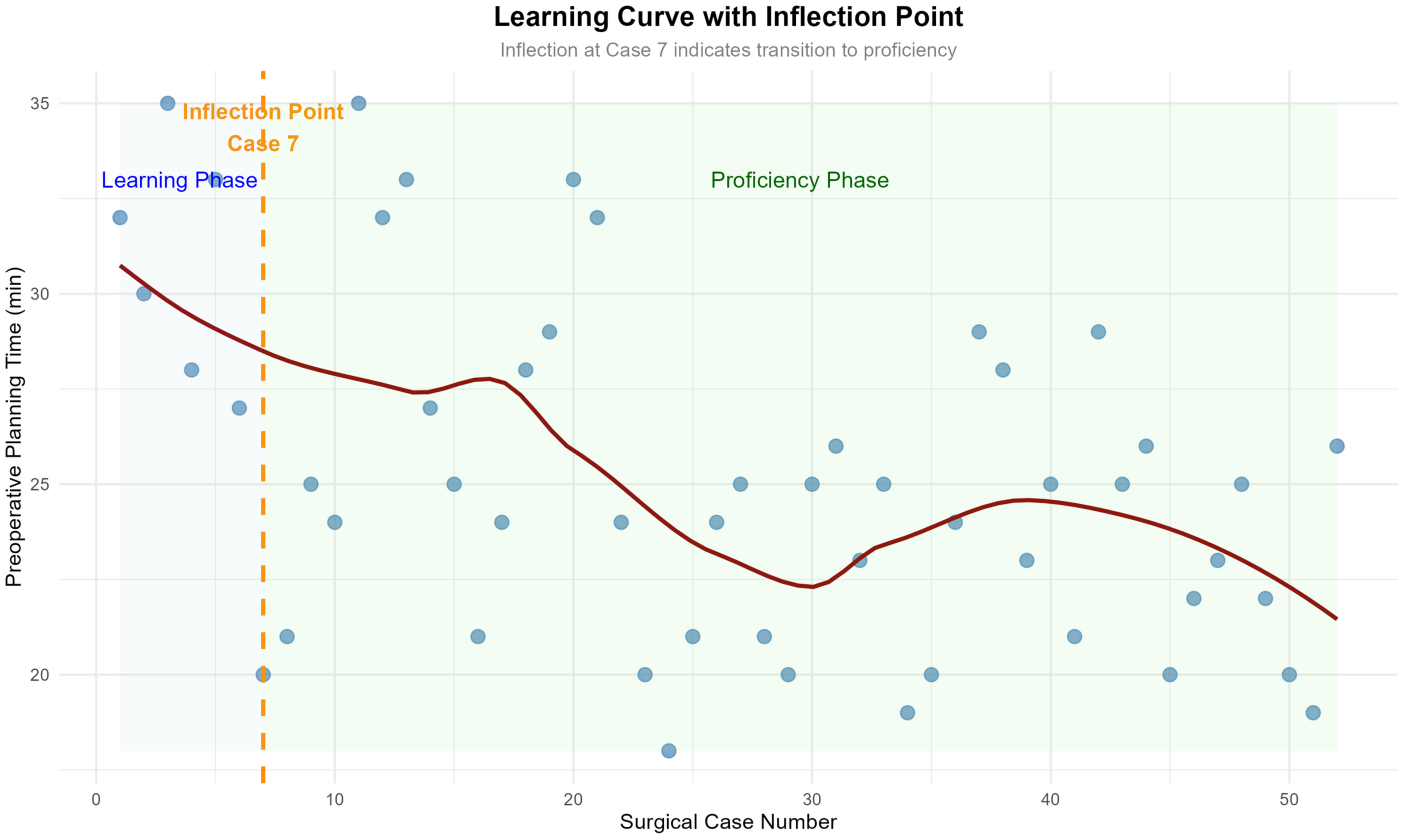
Learning curve with inflection point. The vertical dashed orange line marks the inflection point at Case 7, identified by segmented regression. The shaded areas distinguish the learning phase (blue) from the proficiency phase (green). The solid red line is the LOESS smoothed trend (span = 0.6).

**Figure 4:**
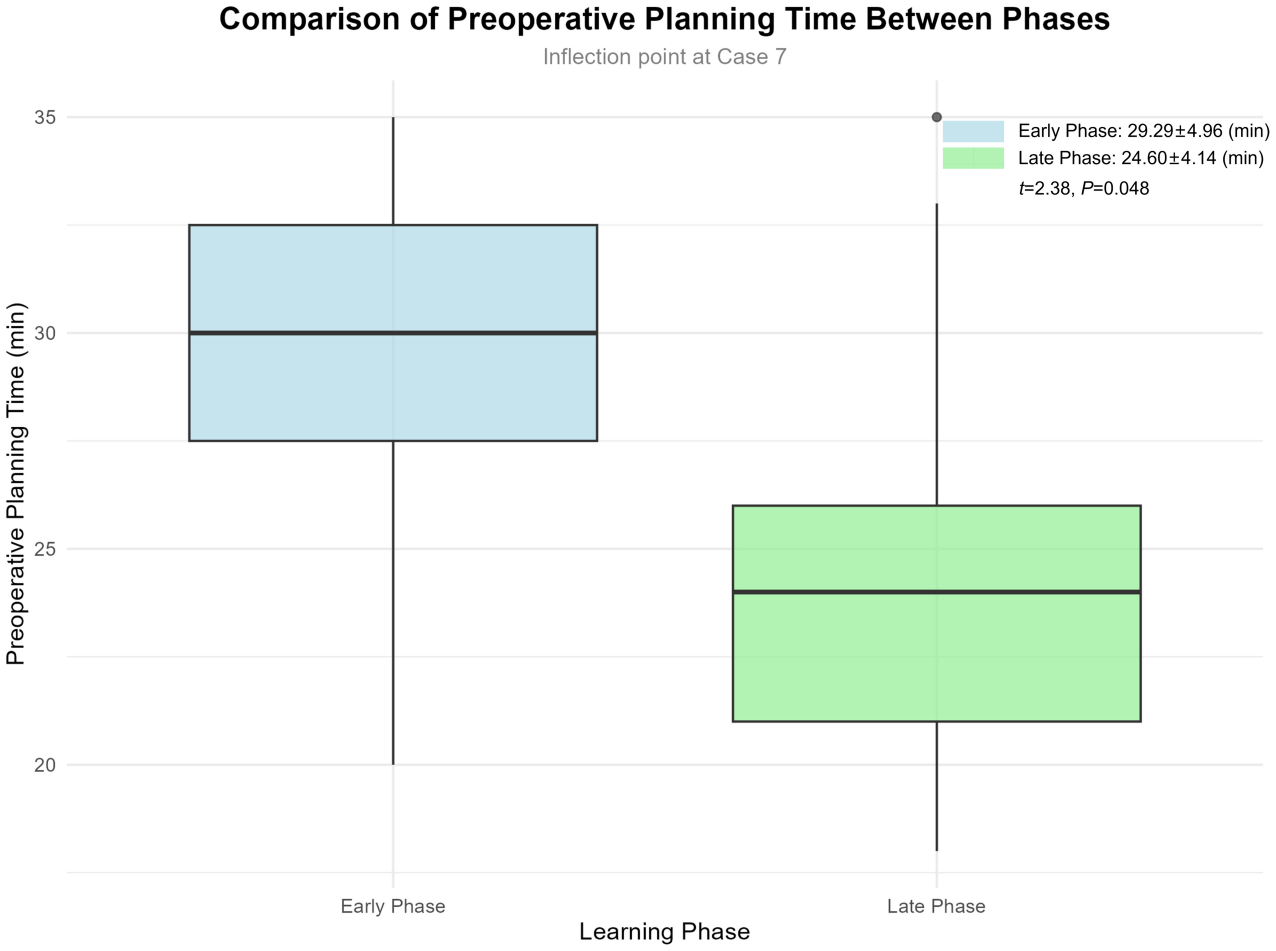
Box plot comparison of preoperative planning time between learning phases. Planning time distribution in the early learning phase (Cases 1–7) vs. the late proficiency phase (Cases 8–52). The box represents the interquartile range (IQR), the horizontal line inside the box indicates the median, and whiskers extend to 1.5 × IQR.

The classic exponential decay model (Time = *a* × Case^∧^(−*b*)) was used to fit the learning curve for quantifying the learning effect. The model equation was determined as Time = 33.04 × Case^∧^(-0.095), with a learning rate *b* = 0.095 (indicating that the preoperative planning time decreased at a rate of 0.095 with the increase in the number of surgical cases). The model's *R*^2^ = 0.233, suggesting that the number of surgical cases explained 23.3% of the variation in planning time, and the remaining variation might be attributed to individual factors such as lesion complexity. Residual analysis revealed a normal distribution (Shapiro–Wilk test: *W* = 0.981, *P* = 0.583). The Durbin-Watson test indicated a slight autocorrelation of residuals (*d* = 1.16, *P* < 0.001), which might be due to the sequential correlation of lesion complexity in consecutive cases. However, the core assumptions of the model were satisfied, and combined with the identification of the inflection point via segmented regression and the significant time difference between phases (*P* = 0.048), it was indicated that the accumulation of the surgical team's experience was associated with improved efficiency of preoperative planning (see Supplementary Figures S1–S3 for details).

## 4 Discussion

This study not only provides evidence supporting the potential efficacy of the individualized localization technology based on 3D Slicer-assisted multimodal image spatial coordinate mapping but also describes a replicable, quantifiable, and rehearsable preoperative planning paradigm. Its core lies in transforming traditional empirical anatomical landmark-based localization into spatial coordinate positioning and physical model rehearsal relying on imaging data. With the 3D visualization model generated by 3D Slicer, surgeons can thoroughly grasp the patient's individualized anatomical structure (such as the relationship between lesion location, blood vessels, and bony landmarks) preoperatively, thereby accurately planning the optimal surgical path (10–12). These technical characteristics correlate with the shorter craniotomy time and higher keyhole positioning accuracy observed in the 3D Slicer group, providing supporting evidence for the core advantage of precise visualization of this technology.

The application of individualized 3D model reconstruction and printing technology is correlated with opportunities for surgical teams to conduct preoperative rehearsals using virtual and physical models, clarifying operational difficulties in advance (29–32). This workflow correlates with reduced intraoperative exploration and time lost to erroneous maneuvers, which is consistent with the lower intraoperative blood loss and postoperative drainage volume observed in the experimental group, reflecting the potential value of preoperative simulation and rehearsal. Precise positioning and clear avoidance of dangerous structures such as venous sinuses correlate with the zero venous sinus injury observed in the experimental group, providing a potential basis for interpreting improved surgical-safety outcomes. The reduced surgical trauma and fewer complications may be associated with faster postoperative recovery, as reflected in the shorter postoperative hospital stay, potentially forming a virtuous cycle where precise positioning supports safety, reduced trauma lowers risks, and rapid recovery correlates with improved prognosis.

In addition, the “shortest safe path” determined through preoperative planning in the experimental group correlates with reduced intraoperative traction or accidental injury to cranial nerves. Meanwhile, the shorter craniotomy time is associated with reduced exposure duration of the surgical field, which may be related to decreased cerebrospinal fluid loss and lower risk of intracranial infection.

A major challenge in suboccipital retrosigmoid approach surgery is the substantial anatomical variability of the transverse sinus, sigmoid sinus, and TSSJ, which manifests in diverse forms (e.g., anteriorly positioned sigmoid sinus, inferiorly located transverse sinus, excessive pneumatization of mastoid air cells) with significant individual differences in the distance from the inner skull plate (4–6, 13–16). Traditional localization methods rely on surface bony landmarks and surgeons' experience, making it difficult to accurately capture these anatomical variations and leading to a relatively high risk of venous sinus injury. In this study, the incidence of venous sinus injury in the control group was 10.3%, and 3 out of the 4 injured patients had anatomical variations such as anteriorly positioned sigmoid sinus or inferiorly located transverse sinus. This result is consistent with the reported incidence of 6%−10% for traditional methods in previous literature (5, 6, 14–18), confirming the limitations of conventional localization techniques.

To address the aforementioned issues, this study proposed and evaluated the 3D Slicer-assisted individualized localization technology based on multimodal image spatial coordinate mapping. This technology can not only capture macroscopic anatomical variations (e.g., deviation in sinus course) but also accurately identify microscopic structural differences (e.g., variations in TSSJ angle, differences in sinus wall thickness). For example, in patients with anteriorly positioned sigmoid sinus, the 3D reconstruction model can directly calculate the shortest distance between the sinus wall and the outer skull plate, avoiding excessive drilling depth caused by “estimation based on surface landmarks” in traditional localization. For patients with excessive pneumatization of mastoid air cells, the model can predict the anatomical association between air cells and venous sinuses, a feature correlated with lower observed risk of cerebrospinal fluid leakage after intraoperative air-cell opening. This advantage suggests that the technology may not merely “improve localization accuracy” but also achieve “individualized precise adaptation” to anatomical variations. It may be particularly suitable for patients with cerebellopontine angle lesions, who have a high incidence of anatomical variations, and correlates with reduced serious adverse sequelae linked to empirical misjudgment in complex variant cases relying upon traditional experiential localization.

For primary hospitals or emergency surgeries where are difficult to deploy rapidly, virtual model navigation using 3D Slicer alone can be employed without the need for physical model printing, taking only approximately 20–30 min. If the hospital is equipped with a 3D printer, the technology can achieve “virtual and physical navigation” through preoperative 3D models, with equipment costs limited to the 3D printer and open-source software, offering higher cost-effectiveness. For complex lesions (e.g., giant meningiomas invading venous sinuses), the technology can serve as a preliminary preoperative planning tool, combined with intraoperative neuronavigation for precise correction to enhance surgical safety. For primary hospitals without 3D printing equipment, cooperation with third-party printing institutions can further lower the barrier to adoption. For complex and difficult cases, cross-hospital collaborative training can be carried out, where large teaching hospitals provide technical specifications for “imaging processing-localization planning” to primary hospitals, addressing the challenge of inadequate proficiency in software utilization at the grassroots level. Additionally, the technology does not rely on high-end imaging equipment (only thin-slice CT+CTV, which is commonly available in primary hospitals), further reducing the hardware threshold for promotion.

Existing precise neurosurgical localization technologies include neuronavigation systems, intraoperative CT/MRI, and robot-assisted surgery, but all have limitations. For instance, neuronavigation systems are extremely expensive (costing several million yuan per unit), making them unaffordable for primary hospitals. Intraoperative CT involves high radiation doses and requires a dedicated operating room. Robot-assisted surgery has complex operational procedures and high equipment maintenance costs (annual maintenance fees of several 100 thousand yuan) (19–23). The technology in this study is not a substitute for high-end navigation equipment but an innovative, low-cost complementary approach.

As an open-source software, 3D Slicer requires no additional equipment purchase costs. Its modular architecture supports functional expansion and compatibility with multiple image formats, making it suitable for promotion and application in medical institutions at all levels (24). Our learning curve analysis provides practical references for the clinical promotion and training of this technology: the inflection point is at the 7th case, and the planning time in the proficiency phase is 16.0% shorter than that in the learning phase with a stable learning curve (*R*^2^ = 0.233). This result correlates with the observation that after standardized training (e.g., modular teaching of “image import-registration-segmentation-marking /3D printing” with a training cycle of 2 weeks), neurosurgical teams are able to integrate 3D Slicer into routine diagnosis-and-treatment workflows, alleviating concerns about workflow disruptions.

However, the technology also has certain limitations: it may face efficiency constraints in emergency surgeries or large-scale clinical applications; software operation requires a certain learning period; and manual segmentation of key structures may introduce certain errors. In the future, artificial intelligence algorithms can be integrated to develop automatic segmentation modules, shortening process time and reducing manual errors. Combining augmented reality (AR)/mixed reality (MR) technology to achieve real-time intraoperative navigation can further improve localization accuracy and operational convenience (7, 12, 21–23, 25–27).

Additionally, the major limitation of this study is the potential selection bias inherent in the retrospective sequential allocation design. Although we strictly unified the inclusion/exclusion criteria, surgical team, and surgical indications for both groups, the sequential implementation of technical protocols may lead to unmeasured confounding factors (e.g., subtle improvements in the surgical team's operative skills over time). However, the study period was relatively short (December 2023–October 2025), and the surgical team was fixed with extensive experience in skull base surgery (more than 10 years for all chief surgeons), which minimized the impact of time-dependent operative skill improvement on the study results. In future prospective randomized controlled studies, we will adopt a random number table method for group allocation to further eliminate selection bias and verify the clinical efficacy of this technology.

Furthermore, although multiple linear regression was employed to adjust for available baseline confounders (i.e., age, sex, lesion type, and anatomical variation), residual confounding cannot be fully excluded. Specifically, the current dataset lacks variables that directly quantify surgical complexity—such as tumor size, lesion depth, and the degree of neurovascular adhesion—which therefore could not be incorporated into the adjustment models. In addition, the number of events for binary safety outcomes (e.g., venous sinus injury and keyhole positioning accuracy) was relatively small, precluding multivariable logistic regression analysis; accordingly, the between-group comparisons for these dichotomous endpoints were based solely on univariable analysis, and the associated estimates should be interpreted with appropriate caution.

Additionally, several considerations regarding localization assessment should be acknowledged. Postoperative localization distance measurements were performed by the same operators conducting preoperative planning, without blinded outcome evaluation. The < 3 mm accuracy threshold was derived from empirical functional neurosurgical robot positioning experience because dedicated reference cut-offs for retrosigmoid keyhole localization are lacking in published literature; actual continuous localization error values in millimeters were not formally summarized in the present analysis. Postoperative CT reconstruction projects were not archived for this retrospective cohort. Furthermore, because this is a retrospective sequential before-and-after cohort study, temporal confounding cannot be fully eliminated; we can only report statistical associations between our localization workflow and perioperative outcomes rather than definitive causal relationships. Although zero venous-sinus injury was observed within the experimental group, the total number of venous-sinus injury events across the whole cohort was limited (only four events in controls), so this safety finding should be interpreted cautiously.

Another notable limitation is that this study only focused on short-term perioperative outcomes, including craniotomy time, intraoperative blood loss, postoperative drainage volume, hospital stay, keyhole positioning accuracy, and venous sinus injury rate, and lacked long-term follow-up data on late complications (e.g., delayed cerebrospinal fluid leakage, chronic intracranial infection, venous sinus stenosis/occlusion) and long-term neurological functional outcomes (e.g., facial nerve, trigeminal nerve function recovery, quality of life of patients). The short-term perioperative indicators can directly reflect the safety and efficiency of the 3D Slicer-assisted localization technology in surgical planning and implementation, but the long-term clinical value still needs to be verified by extended follow-up. Our research team has initiated a long-term follow-up program for all enrolled patients, with a planned follow-up period of 12–24 months, and will further report the long-term outcomes of late complications and neurological function recovery in subsequent studies. In addition, the lack of long-term outcome data is also a common limitation of single-center short-term retrospective studies, and multi-center prospective studies with longer follow-up periods are needed in the future to comprehensively evaluate the long-term clinical benefits of this technology.

Although no venous sinus injury occurred in the experimental group, the sample size is still limited, and this is a single-center study, which may affect the external validity of the results. Future multi-center, large-sample long-term follow-up studies are needed to further verify the application effect of the technology in different populations and lesion types, while focusing on long-term outcome indicators such as postoperative neurological function recovery and quality of life improvement, to provide more comprehensive evidence for the clinical value of the technology.

Based on this core logic, more automated algorithms can be developed or integrated into other navigation platforms in the future to overcome the learning curve and time-consuming issues of current manual operations (23, 24, 28).

## 5 Conclusion

This retrospective sequential before-and-after cohort study confirms that the individualized localization technology based on 3D Slicer-assisted multimodal image spatial coordinate mapping is associated with significantly improved positioning accuracy of the transverse-sigmoid sinus complex in suboccipital retrosigmoid surgery. After adjusting multiple clinical confounders, this workflow correlates with reduced venous sinus injury risk, optimized surgical efficiency and favorable perioperative outcomes. By transforming subjective empirical anatomical judgment into objective quantitative spatial-coordinate data, this method offers a replicable high-precision solution for managing individual skull-base anatomical variations. Featuring low implementation cost and ease of implementation and dissemination, this technology provides evidence-based support for standardized individualized preoperative planning in primary hospitals and possesses broad clinical application prospects. However, prospective multicenter randomized trials with long-term follow-up are still required to further validate its therapeutic efficacy.

## Data Availability

The original contributions presented in the study are included in the article/Supplementary material, further inquiries can be directed to the corresponding author.
