# Supplementary material for: Application of individualized localization technology based on multimodal image spatial coordinate mapping in suboccipital retrosigmoid approach surgery
Source: Front Neurol. 2026 Sep 22;17:1803347. doi: 10.3389/fneur.2026.1803347 (PMC13644167; doi:10.3389/fneur.2026.1803347)
Supplement: Supplementary file 1 [file Data_Sheet_1.docx]

Supplementary Materials

Table S1 Adjusted associations between covariates and perioperative outcomes (multiple linear regression)

| **Covariate** | **Craniotomy time (min)** | | **Intraoperative blood loss (mL)** | | **Postoperative drainage volume (mL)** | | **Postoperative hospital stay (days)** | |
| --- | --- | --- | --- | --- | --- | --- | --- | --- |
|  | Adjusted β (95% CI) | *P* value | Adjusted β (95% CI) | *P* value | Adjusted β (95% CI) | *P* value | Adjusted β (95% CI) | *P* value |
| **3D Slicer group (vs Control)** | −7.20 (−8.60, −5.79) | <0.001 | −31.49 (−43.87, −19.10) | <0.001 | −23.07 (−26.82, −19.32) | <0.001 | −2.60 (−4.18, −1.03) | 0.002 |
| **Age (per year increase)** | 0.02 (−0.08, 0.12) | 0.682 | −0.32 (−1.21, 0.58) | 0.482 | −0.03 (−0.30, 0.24) | 0.827 | −0.001 (−0.11, 0.11) | 0.992 |
| **Female (vs Male)** | −0.82 (−2.26, 0.62) | 0.259 | −7.49 (−20.19, 5.20) | 0.244 | −4.41 (−8.26, −0.57) | 0.025 | −1.74 (−3.35, −0.12) | 0.036 |
| **Lesion type (vs Trigeminal neuralgia)** |  |  |  |  |  |  |  |  |
| **Hemifacial spasm** | −1.94 (−3.66, −0.22) | 0.027 | −5.42 (−20.56, 9.71) | 0.478 | −2.68 (−7.26, 1.90) | 0.248 | −0.90 (−2.83, 1.02) | 0.354 |
| **Meningioma** | −2.13 (−4.31, 0.05) | 0.056 | −16.73 (−35.93, 2.47) | 0.087 | −3.91 (−9.72, 1.91) | 0.185 | −2.02 (−4.47, 0.42) | 0.103 |
| **Acoustic neuroma** | −1.51 (−4.11, 1.09) | 0.252 | 10.27 (−12.65, 33.19) | 0.376 | 3.02 (−3.92, 9.96) | 0.390 | 1.64 (−1.27, 4.56) | 0.266 |
| **Epidermoid cyst** | 0.22 (−2.74, 3.18) | 0.881 | 1.31 (−24.79, 27.40) | 0.921 | 2.04 (−5.87, 9.94) | 0.610 | −1.19 (−4.51, 2.13) | 0.477 |
| **Anatomical variation (vs No variation )** | 7.28 (5.56, 9.01) | <0.001 | 42.50 (27.29, 57.70) | <0.001 | 26.70 (22.10, 31.31) | <0.001 | 3.10 (1.17, 5.04) 3.10 (1.17, 5.04) | 0.002 |

Figure S1 Exponential decay model fit of the learning curve

The exponential decay model (Time = 33.04 × Case^^^(−0.095); solid red line) is fitted to the observed planning time (blue dots). The coefficient of determination (R²) is 0.233, and the estimated learning rate is 0.095.

Figure S2 Cumulative average learning curve of preoperative planning time

The green line represents the cumulative average planning time across consecutive cases. Convergence toward the overall mean time (25.2 minutes; dashed red horizontal line) indicates performance stabilization.

Figure S3 Learning curve of preoperative planning time

Scatter plot of preoperative planning time (minutes) against the case sequence. The LOESS-smoothed trend (span = 0.6; solid red line) shows a general decrease in time with increasing case number, illustrating the learning effect.
